# Reconstruction of Cochlea Based on Micro-CT and Histological Images of the Human Inner Ear

**DOI:** 10.1155/2014/485783

**Published:** 2014-08-03

**Authors:** Christos Bellos, George Rigas, Ioannis F. Spiridon, Athanasios Bibas, Dimitra Iliopoulou, Frank Böhnke, Dimitrios Koutsouris, Dimitrios I. Fotiadis

**Affiliations:** ^1^Institute of Communications and Computer Systems (ICCS), National Technical University of Athens (NTUA), 9 Iroon Polytechniou Street, 15773 Zografou, Greece; ^2^Unit of Medical Technology and Intelligent Information Systems, Department of Materials Science and Engineering, University of Ioannina, 45110 Ioannina, Greece; ^3^First Department of Otolaryngology-Head & Neck Surgery, University of Athens, Ippokrateio Hospital, Vas. Sofias Avenue, 11527 Athens, Greece; ^4^UCL Ear Institute, 332 Grays Inn Road, London WC1X 8EE, UK; ^5^Department of Otorhinolaryngology, Technical University of Munich, Arcisstraße 21, 80333 Munich, Germany

## Abstract

The study of the normal function and pathology of the inner ear has unique difficulties as it is inaccessible during life and, so, conventional techniques of pathologic studies such as biopsy and surgical excision are not feasible, without further impairing function. Mathematical modelling is therefore particularly attractive as a tool in researching the cochlea and its pathology. The first step towards efficient mathematical modelling is the reconstruction of an accurate three dimensional (3D) model of the cochlea that will be presented in this paper. The high quality of the histological images is being exploited in order to extract several sections of the cochlea that are not visible on the micro-CT (mCT) images (i.e., scala media, spiral ligament, and organ of Corti) as well as other important sections (i.e., basilar membrane, Reissner membrane, scala vestibule, and scala tympani). The reconstructed model is being projected in the centerline of the coiled cochlea, extracted from mCT images, and represented in the 3D space. The reconstruction activities are part of the SIFEM project, which will result in the delivery of an infrastructure, semantically interlinking various tools and libraries (i.e., segmentation, reconstruction, and visualization tools) with the clinical knowledge, which is represented by existing data, towards the delivery of a robust multiscale model of the inner ear.

## 1. Introduction

The number of people with hearing impairment is rising mainly due to a growing global population and longer life expectancies. Understanding the exact pathophysiological consequences and mechanisms through which diverse causative factors give rise to hearing impairment in humans requires a thorough understanding of the normal function of the cochlea. Despite significant progress, more work is needed to develop novel approaches to restore hearing [1]. Insight into the pathologic basis of ear disease can be obtained only by postmortem studies of the cochlea and by developing credible animal models. Therefore, finite element models can serve as a powerful platform to study the structure-function relationship in normal and pathological ears and give insights into the planning of novel surgical procedures for the rehabilitation of sensorineural hearing loss.

In order to proceed with the efficient finite element modelling of the active mechanisms of the cochlea, it is essential to reconstruct an accurate three dimensional (3D) model. At the present time, the two main data sources used are histological sections and micro-CT (mCT) images. Rau et al. [2] provide the most comprehensive review on the use of different techniques for the 3D reconstruction of temporal bone images.

The generation and processing of high resolution computed tomography (CT) images* in vivo* allows the exposition of anatomical structures with a maximum resolution of about 0.5 mm, which is not sufficient for the presentation of the fine structures within the cochlea. Regarding the utilization of histological images in the 3D reconstruction of cochlea, different slicing preparation techniques have been used so far for the 3D reconstruction of the cochlea, including histological sections, serial unstained celloidin sections, and tissue block sections [3, 4]. The main advantage of using histology images is its superiority in visualizing soft tissue and individual cellular elements, which are not resolved by micro-CT techniques. The main disadvantage of using histology images is the distorted reconstructions due to the anisotropicity of the acquired images, as well as fixation and staining artifacts. Newer preparation techniques have overcome many of these limitations resulting in more accurate 3D reconstruction by applying feature-based autoregistration algorithms [2].

Shibata and Nagano [5] and later Vogel [6] had a first attempt in middle and inner ear reconstruction using mCT but did not include visualization of the membranous labyrinth, since it was not resolved. The cochlear partition was also not reconstructed as a separate object for further calculations in both cases. Poznyakovskiy et al. managed to visualize the soft tissues in the cochlea of a guinea pig, using mCT, by further staining the specimen with osmium tetroxide (OsO_4_) [7]. They presented a reconstruction of the scala media, but the cochlear partition and the basilar membrane (BM) could not be distinguished and reconstructed. However, Poznyakovskiy et al., recently, presented an algorithm for cochlea segmentation [8], which resulted in the reconstruction of scala tympani. In another approach Shibata et al. tried to visualize the soft tissues in human fetal cochleae using mCT and could show Reissner's membrane and the spiral ganglion [9]. Furthermore, a mCT study was conducted to demonstrate the elevation of the cochlear lumen [10], where a part of the scala tympani and the scala vestibuli was segmented and represented [11].

Coregistration of micro-CT and histological images may be a way forward, as it will be able to combine advantages from both modalities. Attempts of coregistration have already been published for other anatomical regions [12, 13].

## 2. Materials and Methods

### 2.1. Histological Images/Micro-CT Scans

Twenty-six slices of high quality histological images were provided by the University College London Ear Institute (UCL EI).

The mCT images that were used (1452 frames in DICOM format) in the reconstruction were provided by the Department of Otolaryngology of the Technical University of Munich (TUM-Med). The mCT scans were made with a pixel size of 12 mm on the charge-coupled device (CCD) area detector using a cone-beam technology with a 5 mm focal spot X-ray tube (voltage 70 kV, target current 200 mA). The geometrical magnification was 2.034 and thus a spatial resolution of 5.9 mm was obtained. For the reconstruction of 3D geometrical objects the slice thickness was also 5.9 mm and the overall isotropic resolution was 5.9 mm. To minimize noise effects, a long integration time of 4 s was chosen, which yielded a scan time of 36 h for the specimen. The resulting projected data were reconstructed and images with 3400 × 3400 pixels/slice were created. The 3590 slices/specimen produced a large amount of data, that is, 60 Gbyte.

### 2.2. Annotation of Histological Images/mCT Images

Annotation is the generation of regions connected with regard to content by integration according to special criteria describing homogeneity. It is a precondition for surface generation and quantitative determination of volumes. In medical applications, annotation is often performed manually with clinical experts outlining structure contours image by image using pointing devices. This process is time-consuming, and the results often suffer from intra- or interobserver variability. Such limitations are addressed by computerized tools, which, by performing (semi)automatic annotation of medical images, limit user interference and reduce the computational cost. The main challenge in the development of annotation tools is the achievement of sufficient robustness over image variability due to differences in morphology (healthy and impaired anatomic structures) and/or image artefacts (indistinct or disconnected boundaries).

**Figure 1:**
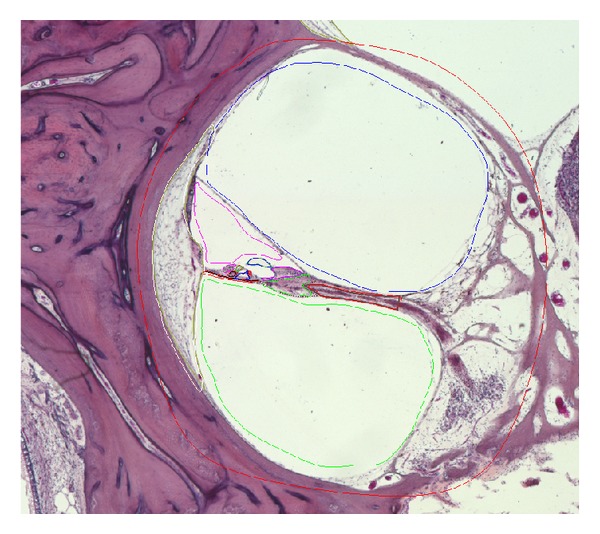
One annotated slice of the histological image provided by UCL-EI.

Points of different structures are annotated manually and corresponding curves are extracted, as depicted in Figure 1. The annotation of the images includes scala vestibuli, scala tympani, scala media, basilar membrane (BM), osseous spiral lamina, organ of Corti, Reissner's membrane, tectorial membrane, inner and outer hair cells, stria vascularis, tunnel of Corti, inner and outer pillar cells, and the subtectorial space.

### 2.3. 3D Segmentation Methodology

Segmentation deals with locating and delineating the boundaries of different (sub)structures of interest and is an essential step towards building 3D models from medical images. Although the segmentation is generally effortless and swift for the human visual system, it can become a highly complex process and considerable challenge for algorithm development. The purpose of 3D segmentation, which is presented in this paper, is the cochlea geometry reconstruction from sets of two dimensional (2D) images corresponding to successive cross-sectional slices of the cochlea. The reconstructed geometry will be used as an input for simulations to generate the mesh and solve the FE problem.

**Figure 2:**
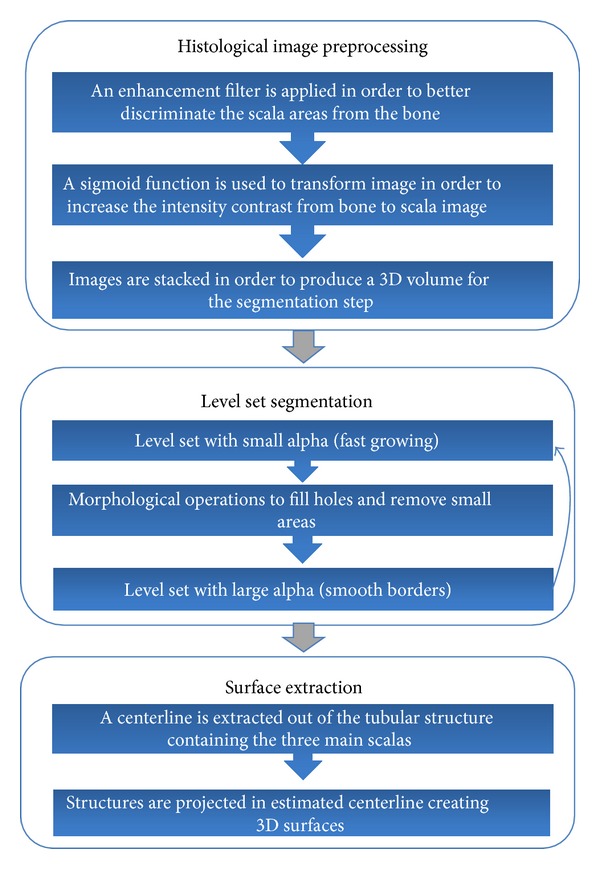
Information flow presenting the main steps of the developed 3D segmentation algorithm.

A hierarchical segmentation approach is followed, developing a hybrid segmentation algorithm, whose steps are presented in Figure 2, consisting of the following.

- *Histological Image Preprocessing.* Large-size structures are first identified and details on smaller structures are gradually obtained, while image enhancement and contrast enhancement filters are applied. At each scale, the image is cropped after segmentation and limited to the relevant structures in an attempt to reduce the computational cost and minimize the possibility to detect unwanted structures at subsequent scales.
- *Level Set Segmentation.* Due to variations in features among the structures of interest, different segmentation methodologies [14] can be used, such as the Hough transform, which is used to detect large structures with distinct boundaries, while deformable models which combine constraints derived from the image data with a priori knowledge about the location, size, and shape of structures are used for structures with less distinct boundaries.
- *Surface Extraction.* The 2D structures identified in the histological images need to be projected on the 3D model derived from the mCT images.

Image segmentation as described above is repeated for the whole set of images. Subsequently, the segmented images are registered through the application of a least-square fitting algorithm, which aims at the minimization of the total volume, to the detected structures. At this point, the distance between successive slices must be known.

## 3. Results

The final reconstructed model exploits the advantages of the histological images (i.e., high quality) as well as the advantages of the mCT images (many frames that provide an accurate 3D overview of the coiled cochlea) by projecting the 2D structures identified in the histological images on the 3D model derived from the mCT images. This projection process includes the following two steps.

1. A centerline is extracted of the tubular structure containing the three main scalas.
2. Structures are projected in estimated centerline creating 3D surfaces.

**Figure 3:**
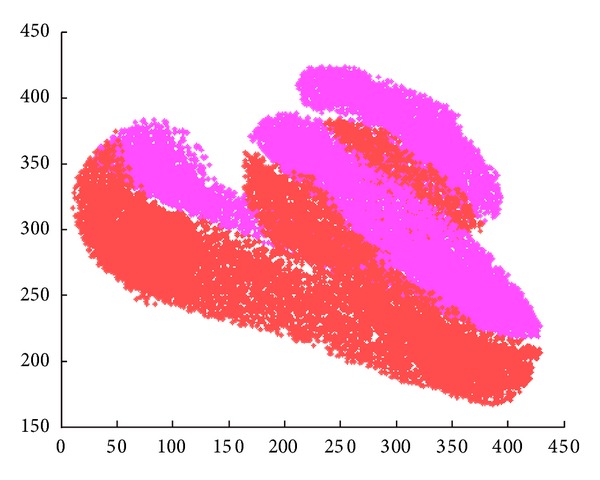
Cloud point corresponding to the two main scalas.

For the extraction of the centerline we used samples from the two main scalas. The samples from the two scalas create a cloud point (Figure 3).

**Figure 4:**
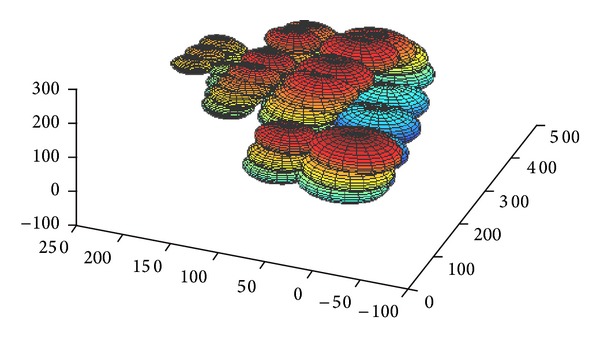
The initial model used for centerline estimation.

**Figure 5:**
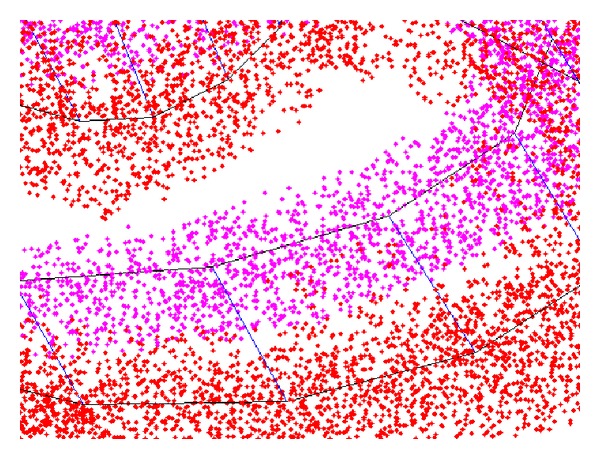
The fitting result. The blue lines connect both the centers of the Gaussian distributions between consecutive mixtures corresponding to the same scala as well as the centers of the mirrored Gaussian distributions corresponding to the two main scalas.

Then the method of [15] is used for estimating the centerline of the cloud point. The fitting method, presented in [15], takes as input an original Gaussian mixture model and given a new sample dataset (derived cloud point) estimates the new means and the transformation of the model using global and local (per mixture) affine transformations. Using this method the geometry of the initial model is maintained in the final model. As an initial model, mirrored spherical Gaussian distributions corresponding to the two scalas are placed across a helix centerline (Figure 4). The final model gives a set of points corresponding to the centerlines of the two scalae as depicted in Figure 5. The average of the two centerlines gives the centerline of cochlea.

**Figure 6:**
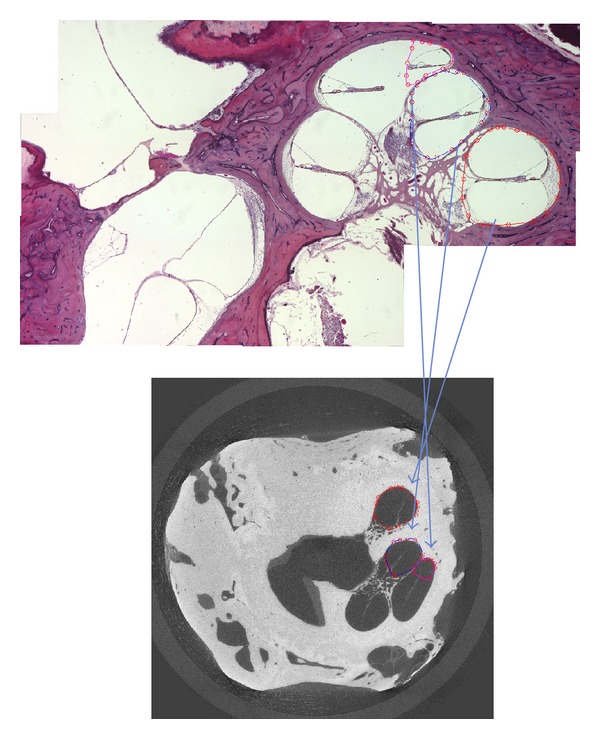
Mapping of a slice of a histological image to a frame of a mCT image.

The structures annotated in the histological images need to be projected on the estimated centerline. Given the control points corresponding to a b-spline interpolation is performed on the centerline and* N* equal distant points are extracted. Furthermore, areas of three different regions on both mCT and histological images were calculated. Scaling was estimated across centerline from maximum to minimum scale using linear interpolation (Figure 6).

**Figure 7:**
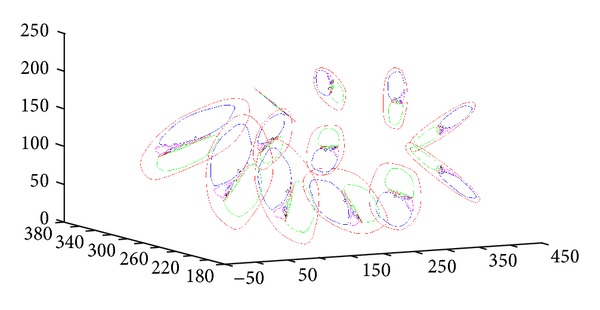
The projection of 2D structures on centerline plane.

Given a centerline point, the* K* points (*x*,* y*) of each 2D structure are projected on the 3D space using the specific point as point of reference, the derivative of the centerline as normal vector, and the scaling estimated previously. Repeating this projection for every a 3D curve (*x*,* y*, and* z*) is created, as depicted in Figure 7.

Finally the 3D curves are merged in a surface corresponding to each structure.

**Figure 8:**
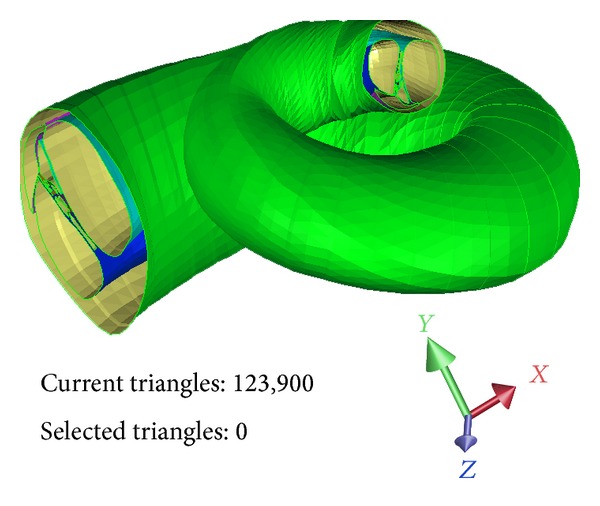
The reconstructed 3D model of the coiled cochlea.

**Figure 9:**
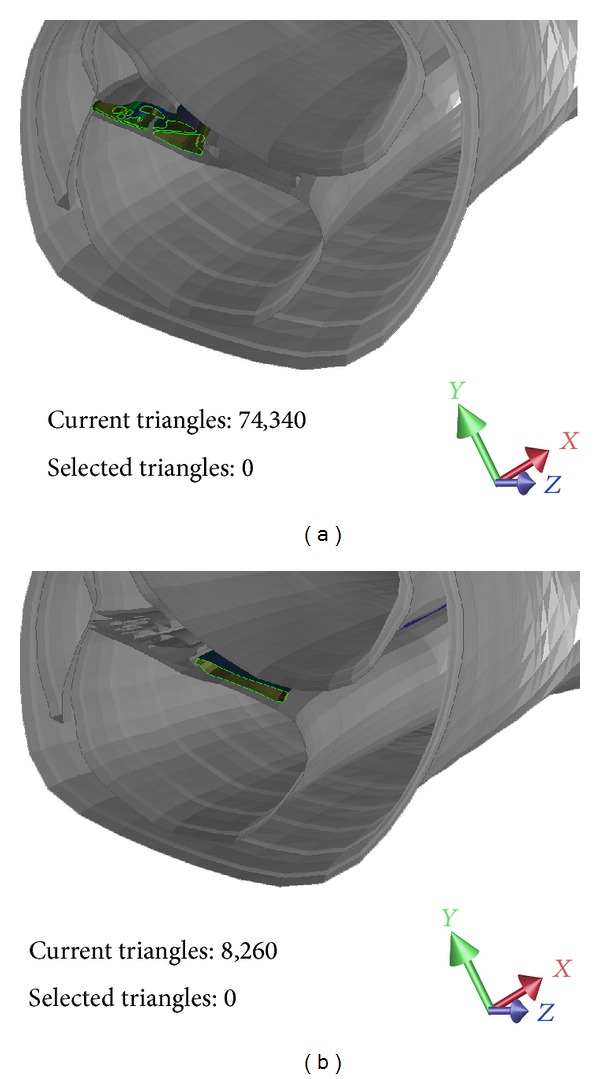
The cochlear partition (a) and the osseous spiral lamina (b).

The results of the hierarchical segmentation and the developed hybrid segmentation algorithm are depicted in Figure 8, while Figure 9 presents a slice in the 3D model depicting the cochlear partition (vibrating part) (Figure 9(a)) and the osseous spiral lamina (nonvibrating part) (Figure 9(b)).

## 4. Discussion

The presented 3D reconstruction study of the cochlea from registration of mCT and histological images provides an anatomical model of the human cochlea, including several parts that are not visible on mCT (scala media, spiral ligament, and organ of Corti) enhancing the accuracy of the 3D model and going a step further towards the efficient studying of a micromechanical model.

The presented reconstruction methodology exploits the advantages of the mCT and histological images and through the registration process eliminates their drawbacks at the segmentation process. The mCT images offer a superior *z*-axis resolution but fail to resolve important soft tissue structures and individual microarchitectural elements (i.e., spiral ligament and organ of corti). On the other hand, histology images are able to resolve in more detail individual cellular structures, but reconstruction is inferior, as only a limited number of images are used. Histological preparations may also suffer from extreme deformations, tears, and destructions.

As a future step in the analysis, instead of using a linear scaling, the structures will be fitted to mCT slices perpendicular to centerline. Also, in the presented work, cochlea structures curves were extracted from a unique histological slice. As a step forward, there will be used curves extracted from different slices on different sites on the centerline and an interpolation between those curves will be performed in order to produce a more accurate reconstruction.

## 5. Conclusions

This new reliable 3D cochlear model demonstrates the basis for further numerical simulations using mathematical procedures, such as the finite element or finite volume method. The development of accurate geometric 3D FE cochlear models will provide a unique opportunity to simulate both normal physiology and cochlear pathology and correlate it to histopathological findings in a wide variety of ear diseases. Additionally it may be used in innovation of novel therapeutic approaches in managing sensorineural hearing loss. These calculations remain the most promising approaches to understand physiological processes and their pathology, which cannot be investigated completely with existing measuring techniques. Although there are studies in the literature that have reconstructed pathological cochleae from CT, MRI, or histological data [12], there are no geometric FE reconstructions of the pathological cochlea for FE modeling purposes.

To this end, the SIFEM project [16] could eventually assist the management of hearing loss. Perhaps one of the most promising and useful aspects of using FEM is the planning of surgical procedures and their predicted effects by employing what-if scenarios. Such scenarios may help in both innovation of surgical techniques and the better design of auditory implants.

## References

[1] Merchant SN, McKenna MJ, Adams JC (2008). Human temporal bone consortium for research resource enhancement. *Journal of the Association for Research in Otolaryngology*.

[2] Rau TS, Würfel W, Lenarz T, Majdani O (2013). Three-dimensional histological specimen preparation for accurate imaging and spatial reconstruction of the middle and inner ear. *International Journal of Computer Assisted Radiology and Surgery*.

[3] Li SF, Zhang TY, Wang ZM (2006). An approach for precise three-dimensional modeling of the human inner ear. *ORL; Journal for Oto-Rhino-Laryngology and Its Related Specialties*.

[4] Wang H, Merchant SN, Sorensen MS (2007). A downloadable three-dimensional virtual model of the visible ear. *ORL*.

[5] Shibata T, Nagano T (1996). Applying very high resolution microfocus X-ray CT and 3-D reconstruction to the human auditory apparatus. *Nature Medicine*.

[6] Vogel U (1999). New approach for 3D imaging and geometry modeling of the human inner ear. *journal for Oto-Rhino-Laryngology and Its Related Specialties*.

[7] Poznyakovskiy AA, Zahnert T, Kalaidzidis Y (2008). The creation of geometric three-dimensional models of the inner ear based on micro computer tomography data. *Hearing Research*.

[8] Poznyakovskiy AA, Zahnert T, Kalaidzidis Y (2011). A segmentation method to obtain a complete geometry model of the hearing organ. *Hearing Research*.

[9] Shibata T, Matsumoto S, Agishi T, Nagano T (2009). Visualization of Reissner membrane and the spiral ganglion in human fetal cochlea by micro-computed tomography. *The American Journal of Otolaryngology—Head and Neck Medicine and Surgery*.

[10] Verbist BM, Ferrarini L, Briaire JJ (2009). Anatomic considerations of cochlear morphology and its implications for insertion trauma in cochlear implant surgery. *Otology and Neurotology*.

[11] Lareida A, Beckmann F, Schrott-Fischer A, Glueckert R, Freysinger W, Müller B (2009). High-resolution X-ray tomography of the human inner ear: synchrotron radiation-based study of nerve fibre bundles, membranes and ganglion cells. *Journal of Microscopy*.

[12] Seise M, Alhonnoro T, Kolesnik M (2011). Interactive registration of 2D histology and 3D CT data for assessment of radiofrequency ablation treatment. *Journal of Pathology Informatics*.

[13] Sengle G, Tufa SF, Sakai LY, Zulliger MA, Keene DR (2013). A correlative method for imaging identical regions of samples by micro-CT, light microscopy, and electron microscopy: Imaging adipose tissue in a model system. *Journal of Histochemistry and Cytochemistry*.

[14] Chan TF, Vese LA (2001). Active contours without edges. *IEEE Transactions on Image Processing*.

[15] Rigas G, Nikou C, Goletsis Y, Fotiadis DI (2013). Hierarchical similarity transformations between Gaussian mixtures. *IEEE Transactions on Neural Networks and Learning Systems*.

[16] Bellos C, Bibas A, Kikidis D SIFEM project: semantic infostructure interlinking an open source finite element tool and libraries with a model repository for the multi-scale modelling of the inner-ear.

